# Air entrapment causing ventricular oversensing early after implantation of an extravascular implantable cardioverter-defibrillator

**DOI:** 10.1016/j.hrcr.2024.05.022

**Published:** 2024-06-05

**Authors:** Julia Hermes, Nadine Molitor, Alexander Breitenstein

**Affiliations:** Cardiology Department, University Hospital Zurich, Zurich, Switzerland

**Keywords:** Mitral valve prolapse, Sudden cardiac death, Implantable cardioverter-defibrillator, Substernal implantable cardioverter-defibrillator, Air entrapment, Oversensing

**Key Teaching Points**

• Mitral valve prolapse (MVP) can be complicated by ventricular arrhythmias and sudden cardiac death. This arrhythmic MVP is defined by the presence of premature ventricular complexes and/or complex ventricular arrhythmias.
• The substernal (extravascular) implantable cardioverter-defibrillator (ICD) represents a novel generation of ICDs where the S-shape lead is placed substernally in the anterior mediastinum.
• Air entrapment is a known phenomenon in nontransvenous ICDs where, early after the implantation, air can surround the sensing electrodes and potentially cause artefacts as well as—in the worst-case scenario—delivery of an inappropriate shock.

## Introduction

Mitral valve prolapse (MVP) is a common disease affecting 2%–4% of the general population. The 2 main underlying etiologies are either a myxomatous change of the mitral valve apparatus (also known as Barlow’s disease) or a fibroelastic deficiency (characterized by chordal thinning and elongation). Mitral annular disjunction is a systolic separation between the ventricular myocardium and the posterior, usually resulting in a loss of mechanical function/support of the posterior mitral annulus. A subset of patients with MVP present with a malignant form (arrhythmic MVP) defined by the presence of MVP (with or without mitral annular disjunction) but with frequent premature ventricular complexes (≥5% total premature ventricular contraction burden) and/or complex ventricular arrhythmias (nonsustained or sustained ventricular arrhythmias, ventricular fibrillation). Prevention of sudden cardiac death in patients with arrhythmic MVP is relatively complex, as no randomized trials exist. While for secondary prevention the indication for an implantable cardioverter-defibrillator (ICD) is given, it is more complex in the primary prevention setting. Previous unexplained syncopes and the presence of high-risk ventricular arrhythmia features support the implantation of an ICD. Whether a transvenous or a nontransvenous device should be implanted does not differ from other types of cardiomyopathies. We present the case of a patient suffering from arrhythmic MVP who underwent the implantation of a nontransvenous ICD whose early postinterventional follow-up was complicated by air entrapment.

## Case report

A 43-year-old woman was diagnosed with arrhythmogenic MVP syndrome. She presented with moderate mitral regurgitation owing to prolapse and thickening of both mitral leaflets as well as a prominent mitral annular disjunction of 10 mm on transthoracic echocardiography, and typical inferior T-wave inversion in the inferior leads on 12-lead electrocardiogram. Both left and right ventricular function were within normal limits. On cardiac magnetic resonance imaging (CMR), prolapse of both mitral leaflets, moderate mitral regurgitation, and mitral annular disjunction of 7 mm were confirmed; the CMR also revealed an area of late gadolinium enhancement at the inferolateral periannular region of the mitral valve apparatus as well as diffuse interstitial fibrosis. Electrically, episodes of nonsustained ventricular tachycardias (VTs) were documented during ambulatory 24-hour Holter electrocardiogram together with a moderate burden (5%) of isolated premature ventricular contractions of polymorphic morphology. Fortunately, the patient’s history was negative for syncopal events, but she suffered from long-standing palpitations. For further evaluation, an electrophysiological examination was performed where, in addition to a typical atrioventricular nodal reentry tachycardia, also polymorphic VTs at a cycle length of 150–180 ms could easily be induced (stimulation with a drive train of 500 ms and 1 extrastimulus at 240 ms without catecholamine injection). In conclusion, according to the ESC consensus document,^1^ 1 high-risk feature (nonsustained VTs) and ≥3 phenotypic high-risk features (inferior T-wave inversions, mitral annular disjunction on echocardiography and on CMR, slightly enlarged left atrium, late gadolinium enhancement on the inferolateral basal segment of the left ventricle) were present and hence, the implantation of an ICD can at least be discussed. Taking this together with the easily inducible polymorphic VT during electrophysiology study, the patient finally decided to undergo the procedure.

**Figure 1:**
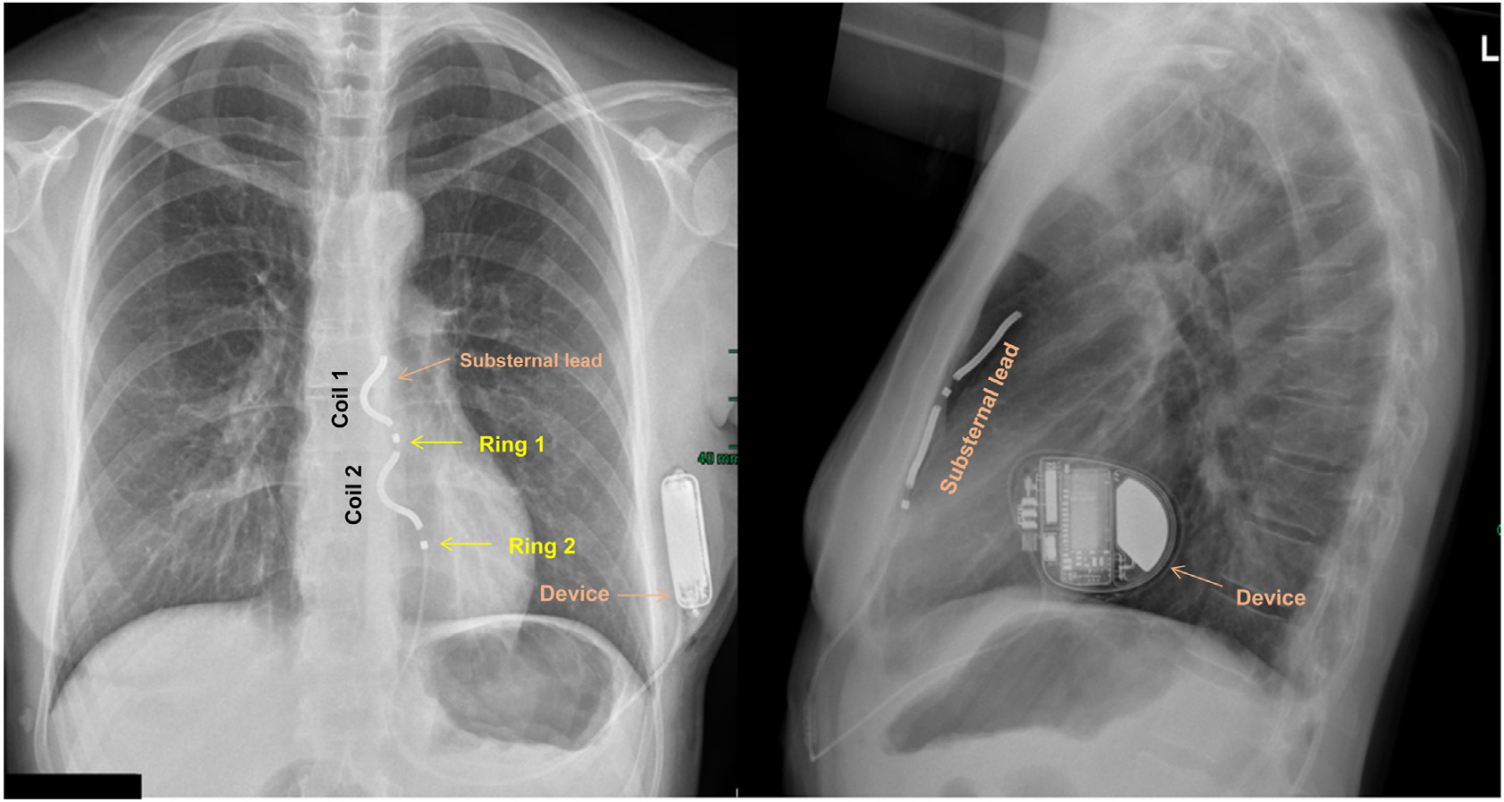
Chest radiograph in posteroanterior (left) and lateral (right) view after successful extravascular implantable cardioverter-defibrillator implantation.

Owing to her young age and the absence of pacing need, the advantages and disadvantages of nontransvenous device options were discussed. The patient chose the option of a substernal extravascular ICD (EV-ICD). The procedure was performed under general anesthesia in the electrophysiology laboratory using fluoroscopy. A chest computed tomography (CT) scan prior to the intervention revealed a distance of more than 1 cm between the posterior border of the sternum and the anterior cardiac structures. Furthermore, the course of both internal mammary arteries was at sufficient distance from the lateral sternal border. Subxiphoid incision was performed at the usual location and the mediastinum entered at the angle between the xiphoid and the left costal margin. Using the angulated introducer tool and biplane fluoroscopic views (anteroposterior and lateral), the 9F sheath was advanced substernally in the anterior mediastinum without any resistance and subsequently the lead was placed at the recommended position (Figure 1). Ventricular sensing (using the standard bipolar vector configuration Ring1-Ring2) was excellent, with an R wave of 5.7 mV without discriminable P wave and an impedance of 342 ohms. Capture threshold in the vector configuration Ring1-Coil2 was 4.8 V @ 2.0 ms and hence comparable to the patients in the pivotal trial (4.9 ± 2.0 V @ 2.0 ms).^5^ Furthermore, defibrillation testing with induction of ventricular fibrillation by a 20 Hz burst was successful at 30 J in the standard vector configuration.

**Figure 2:**
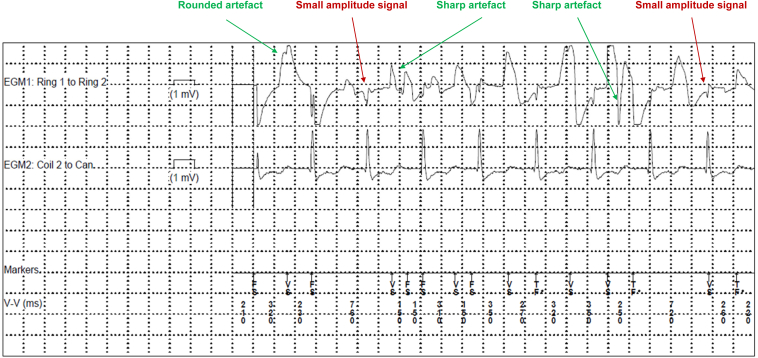
Example of oversensing episode due to air entrapment: Reduced amplitude of the intrinsic signal, sharp artefact signals and more rounded artefacts with a quadrangular signal saturation.

According to our standard procedure after implantation of extravascular ICDs, all antitachycardia treatment options remain deactivated until the next day to ensure stable lead position and that the device is sensing appropriately. The patient felt well after the implantation and only complained about some slight pain at the implantation site. However, the first device interrogation the same day 5 hours after the implantation procedure demonstrated the documentation of several nonsustained ventricular sensing episodes over a period of 10 minutes on bipolar vector configuration Ring1-Ring2 (Figure 2), which occurred 3 hours after the implantation procedure (and 15–20 minutes after the patient changed from a lying to a supine position for lunch). The near-field morphology of these sensed ventricular events showed an erratic behavior with reduced amplitude of the intrinsic signal as well as sharp and rounded artefacts with a more quadrangular signal saturation (Figure 2). Importantly, no such alterations were noted on the simultaneous far-field vector (Coil2-Can) or during the active device interrogation.

**Figure 3:**
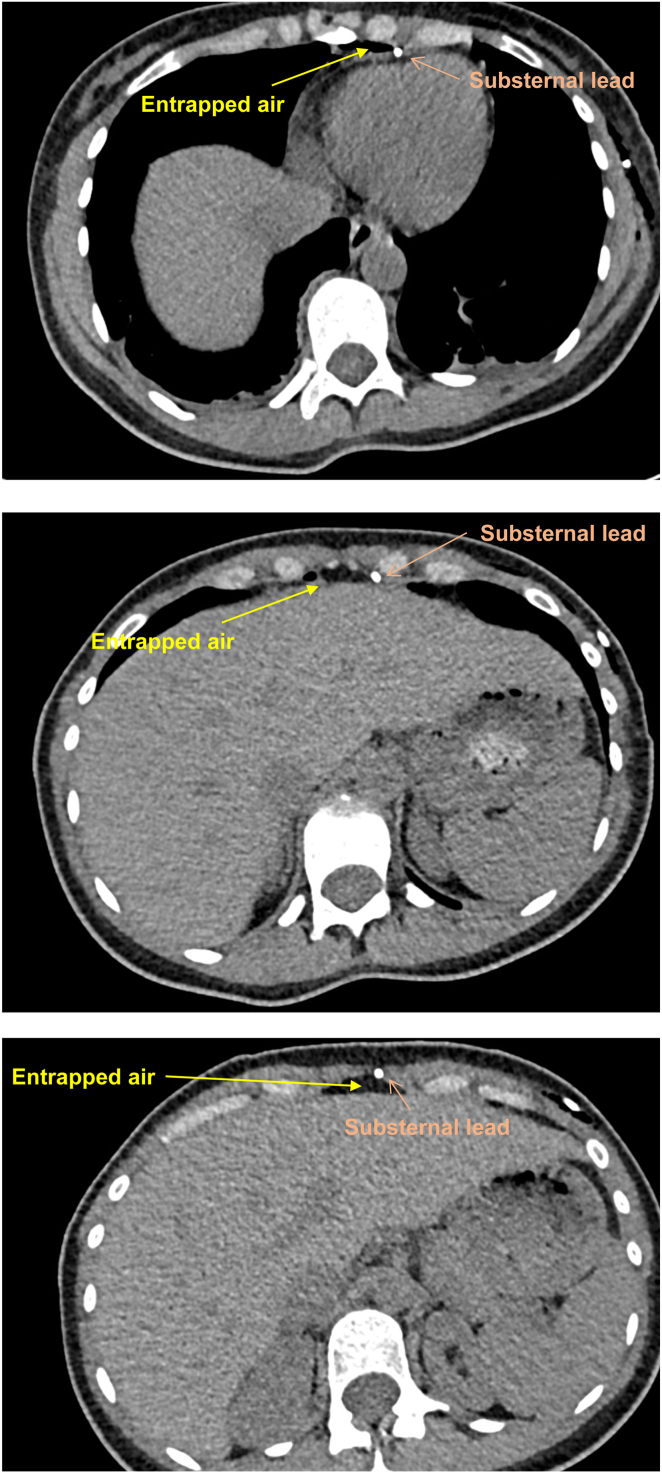
Chest computed tomography scan confirmed the presence of entrapped air surrounding the substernal lead as well as in the tunnel from the subxiphoid pocket.

Air entrapment around one of the bipolar sensing electrodes was the most likely explanation and indeed, a CT scan during follow-up confirmed the presence of air in the substernal tract (Figure 3). As outlined above, the oversensing episode occurred 15–20 minutes after the patient changed to a supine position in bed, and hence it was concluded that owing to upright position of the chest, air from the caudal area had moved cranially to the sensing poles of the lead. During the second device follow-up the day after the initial implantation, no more such episodes were annotated and the device had stable impedance and sensing values (capture threshold was, as usual, not tested again in a non–conscious sedation status). For safety reasons, the antitachycardia therapies remained deactivated until the next day. The patient was discharged in good condition 3 days after the implantation. The 1-month follow-up revealed that no complications or inappropriate treatments had occurred.

## Discussion

The EV-ICD represents a relatively novel approach in preventing sudden arrhythmic death where the high-voltage lead is placed underneath the sternum in the anterior mediastinum and antitachycardia pacing can be delivered via the substernal electrode. Prior to clinical use, this implantation approach underwent a series of preclinical investigations where the possibility of myocardial capture via a catheter^2^ and the success rate of defibrillation with a lead^3^ in the anterior mediastinum were tested. In the ASD2 trial,^4^ the novel S-shape lead configuration was tested for both myocardial capture and defibrillation. Owing to a death as a consequence of inappropriate delivery tool handling, the implantation procedure as well as the tools were adjusted. Finally, the results of the EV-ICD Pivotal Study confirmed the efficacy and safety of this system.^5^ Importantly, there were no instances of mediastinitis or harm to cardiac structures during implantation in the enrolled patients. Prior to implantation, it was recommended that patients undergo chest CT or magnetic resonance imaging scans to investigate the morphology of the heart and extracardiac structures. The implantation procedure and tools were developed based on the input from the above-mentioned preclinical and acute feasibility studies.^3^^,^^4^ Access into the substernal space is gained via blunt finger dissection through the diaphragmatic attachments at the angle of the xiphoid and the left costal margins. During this step, inadvertent introduction of air into the substernal tunnel can potentially not completely be avoided. Furthermore, during removal of the delivery tool while keeping the 9F sheath in the substernal place, the negative pressure within the sheath and its 3-way side arm can lead to air suction. Therefore, it is recommended to connect a saline-filled syringe to the 3-way sidearm of the introducer sheath to avoid inadvertent air suction.

Air entrapment is a known cause of early inappropriate shocks in patients who underwent the implantation of a subcutaneous ICDs (S-ICDs) with a presternal lead placement.^6^^,^^7^ The exact incidence of early inappropriate shocks owing to air entrapment in S-ICDs remains unclear, but the oversensing risk can be present up to 48 hours after the implantation.^8^ The typical alterations of the ventricular sensing as a result of air entrapment consist of (1) artefacts (usually sharp, spike-like, or rounded deflections from the baseline), (2) baseline drift (gradual deviation/wandering of the isoelectric baseline), and (3) reduced QRS voltage.^7^^,^^9^ Entrapped air surrounding the sensing electrode insulates the lead and hence only intermittent tissue contact is present, which leads to artifacts and erroneous oversensing. In addition, the auto-gain function plays another role in ventricular oversensing in cases with QRS amplitude reduction resulting from air entrapment.^8^ Recommendations to avoid air entrapment during the implantation of an S-ICD are the avoidance of digital dissection of the subcutaneous parasternal track and the elimination of air in the parasternal track through normal saline injection and skin massaging.

To the best of our knowledge, this is the first published case of an EV-ICD implantation with subsequent oversensing episodes by air entrapment around the substernal lead. Because of this finding, we support the recommendation from the IDE trial that the device should remain deactivated until the day after the implantation. However, further studies and registry data analyzing the causes for inappropriate shocks in extravascular ICDs with substernal leads are warranted to better understand the causes and mechanisms for inappropriate shocks and to reduce their incidence in the future.

### Limitations

The documentation of entrapped air in this case report is based on only 1 CT scan, and hence, no change of air accumulation over time was shown. But owing to the relatively young age of the patient and the corresponding X-ray exposure, as well as the transient nature of the oversensing episodes (already no longer present during the first device interrogation 5 hours after the implantation), and hence owing to the lack of consequences, it was decided not to perform a follow-up CT scan.

## Disclosures

J.H. has received educational grants from Abbott, Biotronik, and Medtronic. N.M. has received educational grants from Abbott, Biotronik, Boston Scientific, and Medtronic. A.B. has received consulting/presenter fees from Abbott, Bayer Health Care, Biosense Webster, Biotronik, BMS/Pfizer, Boston Scientific, Cook Medical, Daiichi Sankyo, Medtronic, and Spectranetics/Philips.

## References

[1] Zeppenfeld K., Tfelt-Hansen J., de Riva M. (2022). 2022 ESC Guidelines for the management of patients with ventricular arrhythmias and the prevention of sudden cardiac death. Eur Heart J.

[2] Sholevar D.P., Tung S., Kuriachan V. (2018). Feasibility of extravascular pacing with a novel substernal electrode configuration: the Substernal Pacing Acute Clinical Evaluation study. Heart Rhythm.

[3] Chan J.Y.S., Lelakowski J., Murgatroyd F.D. (2017). Novel extravascular defibrillation configuration with a coil in the substernal space: the ASD clinical study. JACC Clin Electrophysiol.

[4] Boersma L.V.A., Merkely B., Neuzil P. (2019). Therapy from a novel substernal lead: the ASD2 study. JACC Clin Electrophysiol.

[5] Friedman P., Murgatroyd F., Boersma L.V.A. (2022). Efficacy and safety of an extravascular implantable cardioverter-defibrillator. N Engl J Med.

[6] Iavarone M., Ammendola E., Rago A. (2023). Air entrapment as a cause of early inappropriate shocks after subcutaneous defibrillator implant: a case series. Indian Pacing Electrophysiol J.

[7] Ali H., Lupo P., Foresti S. (2022). Air entrapment as a potential cause of early subcutaneous implantable cardioverter defibrillator malfunction: a systematic review of the literature. Europace.

[8] Zipse M.M., Sauer W.H., Varosy P.D., Aleong R.G., Nguyen D.T. (2014). Inappropriate shocks due to subcutaneous air in a patient with a subcutaneous cardiac defibrillator. Circ Arrhythm Electrophysiol.

[9] Yang Y.C., Aung T.T., Bailin S.J., Rhodes T.E. (2019). Air entrapment causing inappropriate shock from a subcutaneous implantable cardioverter defibrillator. Cardiol Res.

